# Why do women assume a supine position when giving birth? The perceptions and experiences of postnatal mothers and nurse-midwives in Tanzania

**DOI:** 10.1186/s12884-020-2726-4

**Published:** 2020-01-13

**Authors:** Lilian Teddy Mselle, Lucia Eustace

**Affiliations:** 1 Department of Clinical Nursing, Muhimbili University of Health and Allied Sciences, Dar es Salaam, Tanzania; 2 Department of Nursing and Midwifery, Missenyi District Council, Bukoba, Kagera Tanzania

**Keywords:** Birthing, Birthing position, Midwife, Perceptions, Experiences, Postnatal mothers

## Abstract

**Background:**

Before the advent of Western medicine in Tanzania, women gave birth in an upright position either by sitting, squatting or kneeling. Birthing women would hold ropes or trees as a way of gaining strength and stability in order to push the baby with sufficient force. Despite the evidence supporting the upright position as beneficial to the woman and her unborn child, healthcare facilities consistently promote the use of the supine position. The purpose of this study was to explore the perceptions and experiences of mothers and nurse-midwives regarding the use of the supine position during labour and delivery.

**Methods:**

We used a descriptive qualitative design. We conducted seven semi-structured interviews with nurse-midwives and two focus group discussions with postnatal mothers who were purposively recruited for the study. Qualitative content analysis guided the analysis.

**Results:**

Four themes emerged from mothers’ and midwives’ description of their experiences and perceptions of using supine position during childbirth. These were: women adopted the supine position as instructed by midwives; women experience of using alternative birthing positions; midwives commonly decide birthing positions for labouring women and supine position is the best-known birthing position.

**Conclusion:**

Women use the supine position during childbirth because they are instructed to do so by the nurse-midwives. Nurse-midwives believe that the supine position is the universally known and practised birthing position, and prefer it because it provides flexibility for them to continuously monitor the progress of labour and assist delivery most efficiently. Mothers in this study had no other choice than to labour and deliver their babies in the supine position as instructed because they trusted midwives as skilled professionals who knew what was best given the condition of the mother and her baby.

## Background

The concept of birthing positions as defined by Letushko [1] provides the lens through which different birthing positions are described. The integral pieces of the Letushko concept describe maternal birthing positions as both supine and upright. According to her, maternal birthing positions are influenced by women’s empowerment, age, parity, culture, the biomedical model and the birth attendants who are primary caregivers during the labour process. Maternal birthing positions have a direct effect on the 4PS (Passage, Passenger, Power, and Psyche) which, in turn, affect maternal and infant outcomes [1, 2].

Despite evidence that supports the upright position during labour and delivery as the most optimal way to ensure a positive outcome for the mother and her baby, supine positioning remains the most commonly used by women during childbirth [3, 4]. Not surprisingly, a significant majority of women (68%) gave birth lying on their backs or in a semi-sitting position and a few used other maternal positions [5, 6]. A study in Nigeria reported that 99% of women gave birth in a supine position [7]. It is essential that women are empowered to make personal health decisions, including the choice of birthing position, although health care providers often do not offer this choice. The practice of assuming the supine position for women during childbirth has also influenced home delivery practices, which are known to carry additional risks for the mother due to the involvement of unskilled birth attendants [2, 8]. In Tanzania, there is no specific hospital guideline that informs nurse-midwives’ decision on a labouring woman’s position during birth. Nurse-midwives commonly make decisions for women based on their own beliefs, experiences and training. However, respectful maternity care emphasizes the provision of sufficient and adequate care throughout the birthing process, including the involvement of women as active agents who are fully capable of making their own informed decisions during childbirth [9].

The history of birth positions traditionally used in Tanzania is not well documented. However, Lugina and colleagues [2] have reported that many women who gave birth at home, assisted by traditional birth attendants or relatives, used squatting or other upright positions chosen by the women themselves. Another study conducted elsewhere [10] reported that women in countries not influenced by western cultures, used the upright position when giving birth and that it had a positive labour outcomes. Upright positioning has been reported to be beneficial to both the mother and baby while supine positioning is only beneficial to the midwife/obstetrician and may have adverse effects on the mother and baby [7, 11, 12].

In Tanzania, the supine position had been used consistently despite its adverse effects on maternal and foetal well-being. For example, in 2004, about 80% of women in Tanzania gave birth in the supine position [2]. The adverse effect of this position may include: more painful and less effective uterine contractions, maternal exhaustion due to prolonged labour, reduced pelvic outlet diameters, and reduced blood flow to the uterus, resulting in foetal hypoxia [10, 13]. The purpose of this study was to explore the perceptions and experiences of mothers and nurse-midwives regarding the use of the supine birthing position. More specifically, this article looks at why the supine position is used more frequently during childbirth than other positions even though the evidence discourages it. The findings shall inform policymakers and ensure that health care providers provide evidence-based care during childbirth.

## Methods

### Study design and setting

A descriptive qualitative research design [14] was used to explore postnatal mothers’ and nurse-midwives’ experiences and perceptions regarding the use of the supine position during childbirth. The design enabled the researcher to gain broad and in-depth information from postnatal mothers about their experiences during labour and delivery, particularly in regard to birthing positions [14].

The study was conducted at the Mugana Designated District Hospital (DDH), Missenyi District in Kagera Region. Mugana DDH is the faith-based 140-bed referral hospital for Missenyi district. It serves a population of more than 200,000 in the district and receives patients and clients from nearby regions such as Mwanza, Geita, and Shinyanga. It also serves patients from nearby countries including Uganda. Specifically, this study was carried out in the maternity unit that includes a labour ward, antenatal and postnatal clinics. The labour ward has 7 delivery beds and there is an average of 120 deliveries each month. About100 pregnant women attend the antenatal and postnatal clinics each month for check-ups. There are 36 nurse-midwives working in the maternity unit: 30 of them are on the labour ward and six are in the antenatal and postnatal clinics.

### Participants and recruitment

In order to strengthen the credibility and to better understand the findings, we used two sources of data [14, 15]: postnatal mothers and nurse-midwives. Postnatal mothers were those who had given birth within six months, had more than one normal delivery and could speak Kiswahili. The nurse-midwives were required to work in the delivery room in order to be recruited into the study. We believed that these nurse-midwives would have the most accurate information regarding which birthing positions were used and the reasons for using that preferred position. Recruiting both recent postnatal mothers, as well as midwives working in delivery room, allowed the researchers to explore both participants’ accurate recall of their recent experiences. A purposive sampling strategy was used to recruit participants, ensured the gathering of thorough and in-depth information from the participants who had experience with the birthing positions. The nurse-midwife in charge of the maternity unit was asked to identify postnatal mothers and nurse-midwives who met the inclusion criteria.

After these prospective participants were identified, the researchers approached them and described the aim of the study, the data gathering process and the voluntary nature of their participation. The participants were further informed that there would be no direct benefit for their participation, but that the findings would be used to improve care to women during childbirth. They were also told that they could withdraw their participation at any time, even after having provided consent. Those who agreed to participate were then asked to provide written consent after their questions and concerns were answered.

This study included 23 participants: 16 were postnatal mothers, 4 enrolled nurse-midwives and 3 registered nurse-midwives. In Tanzania, a registered nurse-midwife is a health professional who has successfully completed three years of nursing training at an approved nursing institution and is authorized to practice nursing and midwifery, whereas an enrolled nurse-midwife has completed two years of nursing and midwifery training [16]. Two trained research assistants were recruited to assist the researchers with recording and making observations during focus group discussions. These research assistants were diploma-trained nurses who had had previous experience conducting health-related research.

### Data collection

This study used two methods of data collection: semi-structured interviews (SSI) and focus group discussions (FGD). The integration of semi-structured interviews and focus group discussions provided a better understanding of the use of supine positioning during childbirth from the perspective of postnatal mothers and nurse-midwives and enhanced the credibility of the findings.

### Semi-structured interviews

We conducted seven (7) interviews [17] with nurse-midwives using the SSI guide. The interview guide (Additional file 1) was based on information gained from the literature review and included open-ended questions and probes. The nurse-midwives were asked about their perception of the commonly used positions during childbirth and their experience with other birthing positions. Interviews took place within the hospital premises, in a quiet room that provided privacy from other nurse-midwives and postnatal mothers. All interviews were conducted by the second author (LE), a midwife with experience conducting health research. Each interview was recorded, allowing the researchers to listen later to the interviews and reflect on the interview sessions. The information gathered during the reflection sessions was then used to revise the guide to allow new emerging issues to be included.

### Focus group discussion

Two FGDs with postnatal mothers were conducted within the hospital setting using the FGD guide (Additional file 2). The FGD guide had only one main question followed by probing questions that asked postnatal mothers about their experience using birthing positions and how they chose the birthing position. The questions in the guide were open-ended, which allowed the researcher to explore the perceptions and experiences of postnatal mothers on birthing positions without personalizing opinions. Each FGDs discussion was comprised of eight postnatal mothers who were able to freely discuss their perceptions and experiences on birthing positions. Discussions were conducted in the hospital for convenience reasons. Postnatal mothers came from different areas, therefore, it was not possible to conduct these discussions in the community setting. The first author (LE) moderated the discussions and the research assistants took notes and made observations during the discussions. Before the discussion, ground rules were set whereby postnatal mothers were asked to respect each other’s opinion and were encouraged to actively participate during the discussion. Due to the difficulty of organising and getting an adequate number of postnatal mothers for the focus group discussions within the time allotted for the data collection, only two FGDs were conducted. Nevertheless, group discussions with postnatal mothers yielded adequate information about their experiences with birthing positions and complemented the findings obtained from the semi-structured interviews. Studies [18–20] have recommended that at least two focus group discussions should be conducted to gain saturation if participants have the defining demographic characteristics.

Interviews and focus group discussions took approximately 35 to 60 min and were conducted in Kiswahili, the national language spoken by all participants and researchers. The use of the Kiswahili language during data gathering and the triangulation of the data collection methods and sources increased the trustworthiness of the findings [14]. All interviews and discussions were recorded after the participants provided verbal consent for their conversation to be recorded.

### Data analysis

The inductive method of qualitative content analysis guided the analysis of data [21]. The audio-recorded interviews and discussions were transcribed verbatim into Kiswahili and then translated into English using the semantic translation by the second author (LE) who is fluent in both languages. However, in order to ensure accurate and valid translations [22], the English transcripts were verified by the co-author (LTM) by comparing them with the original Kiswahili transcripts and the audio-recorded interviews and discussions. The researchers discussed any discrepancies between them and then made minor edits to the English transcripts. The researchers analyzed the interviews and discussions in English from the translated transcripts separately. The transcripts were read several times initially in order to gain an understanding of the participants’ perceptions and experiences with birthing positions, and then the meaning units were extracted and carefully condensed without alteration. These condensed meaning units were abstracted and labelled with a code. To ensure adequate translation, all codes and corresponding quotes were reviewed and re-labelled if necessary. Once all codes were identified, they were grouped by participant, either under “postnatal mother” or “nurse-midwife”, and further organized under the themes of 1.Women adopted the supine position as instructed by the midwives, 2. Women experience of using alternative birthing positions, 3. Midwives commonly decide birthing positions for labouring women and 4. Supine is the best-known birthing position. Codes and themes from the content analysis are provided in Table 1.

**Table 1:** Codes and themes from the content analysis of the perceptions and experiences of postnatal-mothers and nurse-midwives

Participants	Codes	Theme
Postnatal Mothers	- I assume the position instructed by the midwife - I was asked to lay on my back - Other positions were not seen well by midwives	Women adopted the supine position as instructed by the midwives
	- I usually see women lie on their backs - I have never used them - Never taught about birthing positions	Women experience of using alternative birthing positions
Nurse-Midwives	- The midwife is the one who knows/chooses the best position - No choice of birthing position for the birthing woman - Midwives decide for the woman which birthing position to use - The midwife needs to educate the mother on the best position - Mothers are not allowed to choose the birthing position they want	Midwives commonly decide birthing position for labouring women
	- Known birth position - Commonly used supine position - Helpful to the woman and the baby - Aids proper observation and delivery - Facilitate quick decision - The woman lay on her back flex legs - Only birthing position taught in schools - The supine position is best as it gives midwife freedom when assisting the woman	Supine is the best-known birthing position

### Ethical considerations

Ethical approval to undertake the study was obtained from the Muhimbili University of Health and Allied Sciences (MUHAS) Research and Ethical Review Board (ref no. MU/PGS/SAEC/Vol.XIV). Further, the District Medical Officer of Missenyi granted permission for data collection. Informed written consent was obtained from each participant after they were informed about the study, assured of the confidentiality of information they provided and the voluntary nature of their participation. All participants were informed that interviews and discussions would be recorded and agreed that their anonymous quotes could be used.

## Results

### Participants’ characteristics

The 7 nurse-midwives interviewed in this study were between 23 and 45 years of age and had worked as nurse-midwives between 1 to 17 years. Four (4) were married and 6 were female. Characteristics of the postnatal mothers are presented in Table 2.

**Table 2:** Demographic Characteristics of Postnatal Mothers

Factor	Category	Participants in the FGDs	
		(*n* = 16)	%
Age at interview	26–32	10	63
	33–38	2	13
	39–44	2	13
	45–50	2	13
Education level	Primary	5	31
	Secondary	1	6
	Above secondary	10	63
Occupation	Housewife	6	38
	Teacher	4	25
	Accountant	1	6
	Warden	1	6
	Secretary	2	13
	Business	1	6
	Soldier	1	6
Number of children	1–2	3	19
	3–4	9	56
	≥5	4	25

### Participants experience and perception of using the supine birthing position

As shown in Table 1, four themes regarding the use of the supine position during delivery emerged: two from postnatal mothers and two from nurse-midwives. The direct quotes from both groups of participants regarding their experiences and perceptions were included to ensure that the findings accurately reflected their accounts.

The term ‘para’ in the postnatal mothers’ identification refers to the total number of pregnancies that a woman has carried past 20 weeks of pregnancy.

### Postnatal mothers perspectives

#### Women adopted the supine position as instructed by the midwives

The supine position is one in which the woman lies on her back with her knees flexed and legs apart, with her feet either supported or not. Postnatal mothers in this study reported using the supine position when giving birth because it was ‘good” and “commonly practised’:

*“…I am used to lying on my back (supine position) that’s what many women are used to. For me to lie on the back it is good because it is not causing any difficulties. I usually see women lying on their backs. This is the best position”.* (Postnatal mother, FGD-1)

Contrary to respectful maternity care principles that emphasize women’s choice of position during birth, the participants’ use of the supine position during childbirth was not their decision. None of the women in this study was given the opportunity to choose the position they preferred during childbirth. Women adopted the supine position as instructed by the nurse-midwives:

*“…when you are about to give birth midwives would ask you to lay by your back (…)”, every delivery, I assume the position instructed by the midwife, and I usually lay on my back (…)”.* (Postnatal mother, 29 years old, para 3)

*“…my experience is to lie on my back; when I arrived at the hospital, I was asked to lie on my back by the midwife, even for previous deliveries I usually lay on my back... Other positions were not encouraged by midwives”.* (Postnatal mother, 28 years old, para 3)

*“…sometimes you can go to the labour room with experience of giving birth, you had three or five deliveries you tell the nurse how you feel, what you want but they can’t listen to you”.* (Postnatal mother, 49 years old, para 6)

#### Women experience of using alternative birthing positions

Studies have reported the advantages of using alternative birthing positions to the mother and her newborn baby [23, 24]. However, it was not common for information about these birthing positions to be included in antenatal health education, despite the fact that some postnatal mothers knew them:

*“…we do not receive any education on birthing positions neither at RCH nor at our homes after marriage, but our friends tell us about different birthing positions which are lateral, squatting and lay on your back.”* (Postnatal mother, FGD-2)

*“ … I only used to lie on my back, other positions I am not used to, but there are other positions. When you talk with other women they say ‘I cannot give birth in laying on my back’, others say ‘I can’t give birth squatting’. So, there are other positions even though we assume only one position”.* (Postnatal mother, 49 years old, para 6)

Some mothers thought that the alternative birthing positions were not good:

*“…I always lied on my back when giving birth, when you are about to give birth midwives would ask you to lay by your back so I think other positions are not good”.* (Postnatal mother, 28 years old, para 3)

It was also learned from this study that mothers who had had the opportunity to use alternative birthing positions had had a very positive experience:

*“… for my first baby, I assumed the lateral position. I felt okay because I lay in the lateral position and I delivered very quickly, I did not spend more hours”.* (Postnatal mother, 29 years old, para 3)

### Nurse-midwives perspectives

#### Midwives commonly decide birthing positions for labouring women

Various factors may influence women’s decision-making during childbirth. These include socio-demographic factors, birth environment, cultural norms, western culture, type of health care provider, level of education and the age of the woman giving birth [2]. It was learned in this study that the decision about which position women should assume when giving birth was commonly made by the nurse-midwives, based on their knowledge and experience. Nurse-midwives believed that they are knowledgeable and competent in birthing practices, including the choice of birth positions. According to our findings, nurse-midwives thought that there would be no point in letting women choose their preferred birthing position:

*“…women are not allowed to choose the birthing position when they are at the hospital. If the woman chooses a position and at last she ends up with the problem, the midwife will be responsible. Why should the midwife allow the woman to choose the position? It is wrong and not the right decision”.* (NM worked in the labour ward for 5 years)

*“…It is difficult to allow her (woman) to use her preferred position because the midwife is the one who knows the best birthing position.* (NM worked in the labour ward for 1 year)

*“… Mothers are not allowed to choose the birthing position they want. I do not know how to say it (pause), maybe it is the midwife knowledge or custom of using the supine position.”* (NM worked in the labour ward for 7 years)

*“…a woman can choose to be in a lateral position, we can allow her in the first stage, but in the second stage, we ask her to be in the lithotomy (supine) position. We do this in order to help her deliver properly”.* (NM worked in the labour ward for 4 years)

It was further learned that nurse-midwives did not assist or advise women to use alternative birthing positions because they themselves did not know these birthing positions:

*“… I cannot say anything on other birthing positions because I have never used them and they are very new to me. However, if there are other positions, then midwives must be taught to help those mothers who will choose other positions”.* (NM worked in the labour ward for 3 years)

*I only know the supine position whereby the woman lies on her back with flex legs and it is the only birthing position taught in schools.”* (NM worked in the labour ward for 5 years)

#### Supine is best-known birthing position

In addition to believing that the supine position is the safest position for delivery, nurse-midwives in this study also thought that it allowed the midwife the ability to assist the woman during delivery most efficiently:

*“…supine position is best; it gives midwife freedom when assisting the woman. It aids the midwife to do the proper observation, for example, to evaluate whether episiotomy is needed or not; you can also identify any abnormalities and make a quick decision in order to assist the mother and her baby. All these cannot be done in other positions.”* (NM worked in the labour ward for 15 years)

*“The supine position is best and safe. Even if she has her preferred position, we convince her to change her mind and use supine”.* (NM worked in the labour ward for 1 year)

*“ … I usually assist women in the supine position. We never conduct delivery in any other positions. The supine position gives support to the midwife while conducting the delivery.”* (NM worked in the labour ward for 5 years)

Despite the fact that the supine position was perceived as the best position to assist women during delivery, nurse-midwives also highlighted the disadvantages associated with the supine position:

*“... It can cause problems for the woman (…) compression of the nerve, which is sensitive and is responsible for the transportation of oxygen and blood if compressed can lead to supine hypotension. If she gets this condition, you can lose her and her baby.”* (NM worked in the labour ward for 17 years)

*“...If there is a delay in 2*^*nd*^ *stage supine position can result in cord prolapsed, foetal distress, severe pain to the woman, maternal distress and a woman may end up with Caesarean section”.* (NM worked in the labour ward for 5 years)

## Discussion

Similar to pregnant women in another study [7], midwives in this study believed that the supine position was the best, most helpful, and best-known birthing position. Most of the midwives believed that the supine position benefits the foetus, mother, and the midwife. They also believed that supine was beneficial to the mother as it made her feel relaxed and allowed her to easily push the baby out because it conserved her strength. Midwives and postnatal mothers also believed that the supine position made it easier for the midwife to conduct deliveries because it allowed them to observe the birth process while assisting the mother during the second stage of labour. These findings are in line with a study done in the United States which concluded that women are encouraged by their health care providers to assume the supine position, in order to allow providers the ability to conduct a delivery more conveniently [25]. Findings of our study are also consistent with a study done in Canada, which revealed that half of the mothers who gave birth in the hospital assumed the supine position [26]. However, researchers have reported that in non-western cultures, in which, parturient mothers were giving birth in an upright position while supported from the back, the supine position was not encouraged [27–29]. This suggests the social nature of giving birth as well as a respect for the physiologic birth process in earlier times.

Midwives and some mothers in this study thought that the supine position is the most advantageous and helpful to the mother. The findings are similar to another study by Okonta [7], which reported that all pregnant women who were surveyed preferred the supine position for childbirth. This preference is contrary to evidence indicating that upright positioning is most beneficial to the mother and infant. Lawrence and colleagues reported that the upright positions such as sitting, squatting, leaning forward or kneeling positions were more often assumed and revealed no any harm to the mother, baby or labour outcome [10]. Other findings from Sweden reported that upright positioning, when assumed in the second stage of labour, is associated with increased sagittal outlet and interspinous diameters compared to supine positioning [24]. A preference for the supine position may be due to a lack of knowledge about its advantages and disadvantages among midwives during their professional training.

Both mother and midwife participants in this study believed that other birthing positions, such as lateral and squatting positions, were not suitable for the birthing woman. Therefore, few postnatal mothers changed birthing positions to ease the pain that they felt while in the supine position. Midwives thought that alternative positions were not good despite evidence that points to increased benefits associated with these positions compared to the supine position. Interestingly, the lateral and the upright positions have been effective in correcting malpositioning of the foetus, contrary to what study participants perceived. In addition, the lateral position has been found to be effective in relieving maternal exhaustion due to prolonged labour and also increases the rate of perineal intactness [23].

Parturient mothers assumed the position preferred by the nurse-midwife attending the delivery. In line with findings from this study, a study done in Canada on the humanization of birth found that mothers were reported not to benefit from decision making in the process of labour [26, 30]. This lack of involvement of women during the birthing process is likely to make some women feel out of control during childbirth and unable to make informed choices [30]. Only one mother in this study reported that she was given an opportunity to choose a position during birth. As reported elsewhere, health care providers made most of the decisions surrounding birth care, which were often quite directive [31]. Another study [11] found that the nurse-midwives’ choice of birthing position for the parturient mothers was based on their custom. Nurse-midwives chose the position that was easiest for them to carry out their professional procedures and the parturient mother was left passive. Searle [32] reported that health care professionals, including midwives, significantly influence birthing positions of parturient mothers. Childbirth had historically been viewed as an illness rather than a natural process. Consequently, this historical construction has reduced mothers to the role of the passive recipient rather than active participant in decisions regarding personal health information [28]. Elsewhere, researchers have reported that if mothers are allowed to choose their preferred birthing position, they would assume alternative birthing positions [5].

Consistent with other studies [33], the nurse-midwives in our study reported that mothers who had delivered several times or had had previous deliveries in the village challenged them by insisting on assuming birthing positions other than supine. This attitude of the mothers was perceived negatively by nurse-midwives, who termed it ‘uncooperative,’ as the mothers’ wishes contradicted the views of the nurse-midwives. The nurse-midwives responded by encouraging them to assume the supine position instead, as taught during their training [28]. Another study reported that mothers who gave birth at home with traditional birth attendants retained control over their birthing positions, while at the hospital, they feared unfamiliar birthing positions, including the supine position [34]. In Bangalore, mothers who maintained an upright position (supported sitting position) in the second and third stages of labour also challenged health care professionals to promote evidence-based practices by shifting away from using the routine supine position during delivery [35].

When women are empowered to make their own choices about their birth process, including birth position, it helps to build confidence and promote autonomy. It is imperative that the birthing woman is respected and that her choices are supported with unbiased information and evidence about best practices [36, 37]. A study in Malawi reported significant improvement in parturient mothers’ overall satisfaction with their care simply because they were informed about the different birthing positions and given a choice [38]. To further support this, another study in Ghana revealed that parturient mothers appreciated a competent midwife who acted not only as a care provider but also as a labour companion, thereby empowering mothers to take the lead while also receiving relevant information from the midwife. Informing women about different birthing positions is one way of promoting the empowerment of mothers and has been found to positively influence the perceptions of mothers toward the birthing process [36]. Overall, shared decision-making brings positive experiences for both parturient mothers and their health care providers and increases mothers’ satisfaction with the labour process [39].

This study revealed that midwives primarily conducted deliveries using only the supine position. Most nurse-midwives and postnatal mothers did not know alternative birthing positions. Nurse-midwives also acknowledged not being able to conduct deliveries using alternative birthing positions given their lack of experience as well as knowledge. Their primary concern was feeling incompetent to assist mothers in non-supine births due to insufficient training during their professional courses as well as the increased pressure that comes with potential obstetric emergencies. As indicated in the International Federation of Gynecology and Obstetrics (FIGO) guidelines, increased competency of health care providers and adequate equipment/space would facilitate the choice of birthing position by mothers. The use of the supine position during birth has been promoted through midwifery training and has now become a ‘habit’ that continues to be practised without evidence to support it as the ‘best’ birthing position [13, 32, 36].

Throughout the study, it became evident that midwives’ practice of assisting women to use the supine position during birth was greatly influenced by the biomedical model. Midwives, much like their physician colleagues, often believed that every woman who seeks birth care at a health facility is “sick” and lacks knowledge concerning health care or treatment. Based on this assumption, women are rarely provided with a chance to choose their birthing position during labour and delivery. Moreover, birth is frequently dominated by interventions that hinder the natural progress of childbirth [40]**.** The practice of not giving women any choice in their own birthing process may influence women’s birth-seeking behaviours, as many women in Tanzania still give birth at home assisted by unskilled personnel [41].

Nurse-midwives often take the lead of the birthing process, which should include a choice of birthing position used by the birthing woman. In order to promote choice, autonomy, and empowerment of the mother, midwives must actively provide women with information concerning alternative birthing positions. Holistic care must be a priority while caring for a mother in labour. Cooper [28] highlights that the individualized, holistic care provided by community midwives should be translated into health care facilities as a way of improving person-centred care [40]**.**

Some postnatal mothers in this study reported knowing different birthing positions and others reported using them in their previous deliveries. However, midwives were resistive to accepting mothers’ choices, especially if alternative positions were mentioned. These findings are inconsistent with a study done in Sweden that outlined how health care providers must promote evidence-informed care and centre the mother as an agent in her care to improve perceptions of mothers’ on the birthing experience [37]. In this study, midwives’ reasons for not promoting alternative birthing positions included the belief that they were unsafe for the mother and the foetus, and not favourable to the management of labour for midwives. In addition, midwives in Tanzania are not taught alternative positioning in their training. As a result, mothers had little room to choose their preferred birthing position due to their view that the midwives were the experts and mothers in childbirth needed to follow their directives.

### Study limitations

While this study was conducted with rigour and provides important insights into the perceptions and experiences of women and midwives on birthing positions, the difficulties involved in translating the interviews should be acknowledged. The analysis of the semi-structured interviews and FGDs were completed in English from translated transcripts. The transcripts were verified by co-authors fluent in Kiswahili to ensure accurate translations, and all codes and themes were discussed among the researchers by reviewing the original Kiswahili transcripts. Additionally, after every interview, researchers had the opportunity to listen to the audio-recorded interviews, reflect on the interview sessions and gather additional information in the reflection sessions, all of which were used during analysis to verify the interpretation of themes.

Postnatal mothers were interviewed within the hospital setting far from the postnatal ward. Having interviews in the hospital setting may influence how mothers reported their experience of childbirth. However, all attempts were made to ensure confidentiality and study participants were informed that their responses would not be shared with their healthcare providers. The aim of this study was not transferability of findings, but rather to gain an understanding and shed light upon why the supine position is commonly assumed by women during childbirth in the health care facilities in Tanzania, despite the fact that other and better alternative positions exist.

## Conclusion

The experience of women in Tanzania around the use of the supine position during childbirth seems to be a direct result of the knowledge and professional experience of their midwives. The women were commonly asked by midwives to assume the supine position despite having had experiences of using other birthing positions in their previous delivery. Midwives preferred the supine position because they thought it was the safest position because it facilitated the monitoring of the woman and her unborn baby during labour and delivery. Women had to assume the supine position as instructed by the nurse-midwives because they trusted them as the experts and skilled professionals who knew what was best for the mother and baby. Moving forward, midwives need to integrate the principles of respectful maternity care during birth so that women are provided with the opportunity to choose a position that they think is suitable for them. Future research needs to examine the factors that would enable women to have choices regarding their birth experience and to take control of their birth process.

## Supplementary information

**Additional file 1.** Semi-structured Interview Guide for Nurse-midwives.

**Additional file 2.** Focus Group Discussions (FGD) Guide for Postnatal Mothers.

## Data Availability

The data and material are available from the corresponding author on reasonable request.

## Abbreviations

4PS: Passage, Passenger, Power, and Psyche
FGD: Focus Group Discussions
FIGO: International Federation of Gynaecology and Obstetrics
MDDH: Mugana Designated District Hospital
MOHCDGEC: Ministry of Health Community Development, Gender, Elderly and Children
MUHAS: Muhimbili University of Health and Allied Sciences
NM: Nurse-Midwife
RCH: Reproductive and Child Health
SSI: Semi-Structured Interviews
