# Supplementary material for: Why do women assume a supine position when giving birth? The perceptions and experiences of postnatal mothers and nurse-midwives in Tanzania
Source: BMC Pregnancy Childbirth. 2020 Jan 13;20:36. doi: 10.1186/s12884-020-2726-4 (PMC6958681; doi:10.1186/s12884-020-2726-4)
Supplement: Supplementary file 1 — Additional file 1. Semi-structured Interview Guide for Nurse-midwives. [file 12884_2020_2726_MOESM1_ESM.docx]

**Semi-structured Interview Guide for Nurse-midwives**

1. Please, could you tell me what is your perceptions regarding birthing positions?

Probe….

- Who chooses the position a woman should assume during delivery? Why?
- What are the women’s preferred birthing positions?
- What reasons do they give for those preferred positions?
- What is your opinion regarding the supine position?

1. What is your experience of assisting deliveries in alternative birthing position?

Probe….

- Any difficulties associated with alternative birthing positions?
- Can you please explain any obstacles women encounter when using alternative birthing positions?
- What do you think should be done to promote alternative birthing positions?

**Thank you**
