# Supplementary material for: Why do women assume a supine position when giving birth? The perceptions and experiences of postnatal mothers and nurse-midwives in Tanzania
Source: BMC Pregnancy Childbirth. 2020 Jan 13;20:36. doi: 10.1186/s12884-020-2726-4 (PMC6958681; doi:10.1186/s12884-020-2726-4)
Supplement: Supplementary file 2 — Additional file 2. Focus Group Discussions (FGD) Guide for Postnatal Mothers. [file 12884_2020_2726_MOESM2_ESM.docx]

**Focus Group Discussions (FGD) Guide for Postnatal Mothers**

1. Please, can you tell me your experience of using the birthing positions?

Probe ……

- What are the common birthing position women assume during delivery? why?
- How the decision is made about the birth position a woman should assume?
- What is your opinion regarding the supine position?
- What is your experience of using other birthing positions?

**Thank you**
